# Pharmacokinetic interaction of ketoconazole, clarithromycin, and midazolam with riociguat

**DOI:** 10.1186/2050-6511-14-S1-P5

**Published:** 2013-08-29

**Authors:** Corina Becker, Reiner Frey, Sigrun Unger, Dirk Thomas, Michael Reber, Gerrit Weimann, Hartmut Dietrich, Erich R Arens, Wolfgang Mueck

**Affiliations:** 1 Clinical Pharmacology, Bayer HealthCare Pharmaceuticals, Wuppertal, Germany; 2 Global Biostatistics, Bayer HealthCare Pharmaceuticals, Wuppertal, Germany; 3 ClinPharmCologne, MEDA Manufacturing GmbH, Cologne, Germany

## Background

Riociguat, an oral soluble guanylate cyclase stimulator, is under investigation for pulmonary hypertension treatment. Cytochrome P450 (CYP)-mediated oxidative metabolism is one of the major riociguat clearance pathways. The pharmacokinetic interactions between riociguat and ketoconazole (multi-pathway CYP and P-glycoprotein/breast cancer resistance protein [P-gp/BCRP] inhibitor), clarithromycin (CYP3A4 inhibitor), and midazolam (CYP3A4 substrate) were investigated.

## Methods

Three open-label, randomized, crossover studies were performed in healthy males. In the first study, subjects received riociguat 0.5 mg ± ketoconazole (4-day pretreatment with once-daily [od] ketoconazole 400 mg, then riociguat + 1 dose of ketoconazole 400 mg) (n=16). In the second study, subjects received riociguat 1 mg ± clarithromycin (4-day pretreatment with twice-daily clarithromycin 500 mg, then riociguat + 1 dose of clarithromycin 500 mg) (n=14). In the third study, subjects received three-times daily (tid) riociguat 2.5 mg for 3 days, then 1 day of riociguat 2.5 mg tid ± midazolam 7.5 mg (n=24). Pharmacokinetic parameters, safety, and tolerability were assessed.

## Results

**Figure 1:**
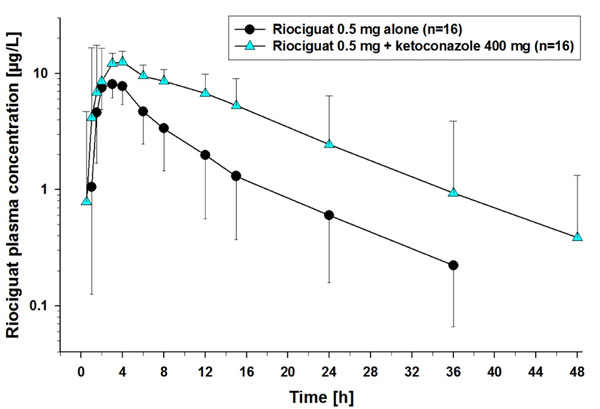
Plasma concentrations of riociguat 0.5 mg alone or in combination with ketoconazole 400 mg.

**Figure 2:**
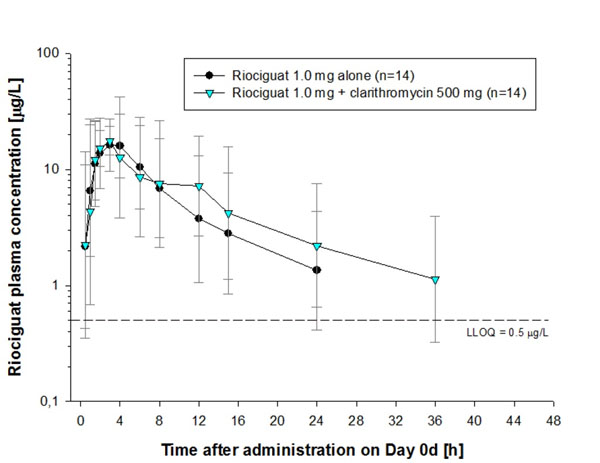
Plasma concentrations of riociguat 1 mg alone or in combination with clarithromycin 500 mg.

**Figure 3:**
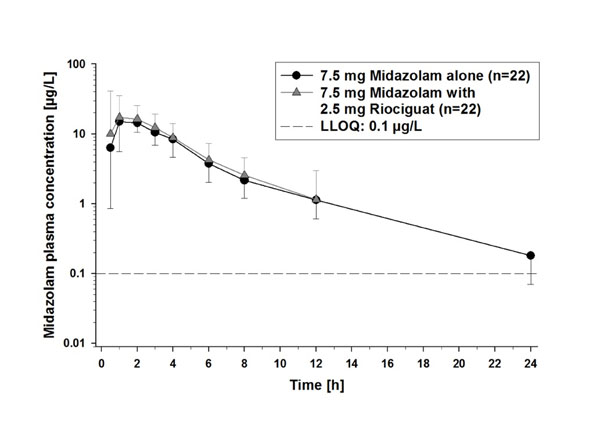
Plasma concentrations of midazolam 7.5 mg alone, and in combination with riociguat 2.5 mg. LLOQ, lower limit of quantification.

Pre- and co-treatment with ketoconazole increased riociguat mean AUC by 150% and mean C_max_ by 46% (Figure 1; Table 1). Pre- and co-treatment with clarithromycin increased riociguat AUC by 41% without significantly increasing C_max_ (Figure 2; Table 1). Riociguat pre- and co-treatment did not significantly alter the AUC or C_max_ of midazolam (Figure 3; Table 2). In the ketoconazole study, adverse events (AEs) were reported in 4 (25%), 6 (38%), and 5 (31%) subjects treated with riociguat alone, riociguat + ketoconazole, and ketoconazole alone, respectively. In the clarithromycin study, AEs were reported in 4 (29%), 9 (64%), and 9 (64%) subjects treated with riociguat alone, riociguat + clarithromycin, and clarithromycin alone, respectively. In the midazolam study, AEs were reported in 20 (87%), 11 (48%), and 6 (27%) subjects treated with riociguat alone, riociguat + midazolam, and midazolam alone, respectively. The most common AEs with riociguat ± ketoconazole, clarithromycin, and midazolam across the three studies were headache and dyspepsia. One serious AE was reported in the midazolam study (elevated creatine phosphokinase; not drug-related).

**Table 1:** The effects of ketoconazole and clarithromycin on riociguat pharmacokinetics (geometric means and coefficients of variation)

	Riociguat/ketoconazole study				Riociguat/clarithromycin study			
	
Parameter	Riociguat 0.5 mg (n=16)		Riociguat 0.5 mg + ketoconazole (n=16)		Riociguat 1 mg (n=14)		Riociguat 1 mg + clarithromycin (n=14)	
	
	GM	%CV	GM	%CV	GM	%CV	GM	%CV
AUC (µg·h/L)	81.9	78.6	204.9	44.9	171.1	97.0	240.0	88.9
C_max_ (µg/L)	9.4	29.9	13.7	19.3	20.8	37.7	21.6	33.9
t_1/2_ (h)	7.3	78.5	9.2	57.1	6.4	77.1	7.9	54.6
CL/f (L/h)	6.1	78.6	2.4	44.9	5.8	97.0	4.2	88.9

AUC, area under plasma concentration–time curve; CL/f, total riociguat clearance from plasma; C_max_, maximum riociguat plasma concentration; CV, coefficient of variation; GM, geometric mean; t_1/2_, elimination half-life.

**Table 2:** The effects of riociguat on midazolam pharmacokinetics (geometric means and coefficients of variation)

Midazolam/riociguat study				
	Midazolam (n=22)		Midazolam + riociguat 2.5 mg (n=22)	

Parameter	GM	%CV	GM	%CV

AUC (µg·h/L)	91.1	34.3	98.2	37.0
C_max_ (µg/L)	29.0	45.1	29.5	41.5
t_1/2_ (h)	4.5	35.9	4.3	34.9

AUC, area under plasma concentration–time curve; C_max_, maximum riociguat plasma concentration; CV, coefficient of variation; GM, geometric mean; t_1/2_, elimination half-life.

## Conclusions

The combined use of riociguat with multi-pathway inhibitors such as anti-mycotics (eg ketoconazole) or HIV protease inhibitors should be avoided due to the expected increase in riociguat exposure. General dose adaptation for patients with co-medication inhibiting the CYP3A4 pathway or the P-gp/BCRP-mediated excretion of riociguat, beyond the dose titration concept for riociguat, is not deemed necessary. Riociguat ± ketoconazole, clarithromycin, or midazolam was generally well tolerated.

